# Epistemic justice in public involvement and engagement: Creating conditions for impact

**DOI:** 10.1111/hex.13553

**Published:** 2022-06-30

**Authors:** Kristin Liabo, Emma J. Cockcroft, Kate Boddy, Leon Farmer, Silvia Bortoli, Nicky Britten

**Affiliations:** ^1^ Institute for Health Research University of Exeter Medical School Exeter UK; ^2^ Peninsula Public Engagement Group (PenPEG), NIHR ARC South West Peninsula, Institute for Health Research University of Exeter Medical School Exeter UK; ^3^ Living Systems Institute University of Exeter Exeter UK

**Keywords:** boundary objects, collaboration, epistemic justice, experiential knowledge, knowledge boundaries, public involvement

## Abstract

**Introduction:**

Patient and public involvement in research is anchored in moral and epistemological rationales. Moral rationales relate to the public having a right to influence how knowledge about them is generated. Epistemological rationales relate to how research design and implementation can improve when informed by experiential, as well as technical, knowledge. In other words, public involvement can increase the epistemological resources of researchers, and contribute to research that is fit for purpose and has high external validity.

**Methods:**

This article presents an analysis of 3 meetings and 11 interviews with public collaborators and researchers in three UK‐based health research studies. Data comprised transcripts of audio‐recorded research meetings and interviews with public collaborators and researchers. Data were first analysed to develop a data‐informed definition of experiential knowledge, then thematically to investigate how this experiential knowledge was considered and received within the research space.

**Results:**

At meetings, public collaborators shared their experiential knowledge as stories, comments, questions, answers and when referring to their own roles. They were aware of crossing a boundary from everyday life, and some adapted their contributions to fit within the research space. Although researchers and public collaborators made efforts to create an inclusive climate, obstacles to impact were identified.

**Conclusions:**

Considering experiential knowledge as a boundary object highlights that this knowledge has a different form to other kinds of knowledge that contribute to research. To enable impact from experiential knowledge, researchers need to create a space where public collaborators experience epistemic justice.

**Patient and Public Contribution:**

The Peninsula Public Engagement Group (PenPEG) was involved in the planning and conceptualization of the study, including the development of the ethics application and the interview schedules. One member of this group (Richard Fitzgerald) and one from outside the group (Leon Farmer), were full members of the author team and were involved in the data analysis. Leon Farmer has since become a member of PenPEG. Richard Fitzgerald and Leon Farmer were not involved in the three research studies sampled for this study. Sadly Richard Fitzgerald died during the course of this study.

## 1 INTRODUCTION

‘Patient and public involvement’ (PPI) refers to forms of partnership and collaboration with patients and members of the public in research. We here use the term ‘public collaborator’ when referring to members of the public or patients who are involved.

PPI arose partly as a response to flawed research. For example, Oliver^1^ exposed how assumptions about disability amongst able‐bodied researchers skewed survey questions designed to understand the lives of disabled people, rendering the results invalid. Another argument is that research is wasted when the needs of potential users are ignored.^2^ Involvement can increase the epistemological resources available to researchers, by pointing out gaps in their knowledge or sharing how health is experienced by patients and carers.

‘Experiential knowledge’ is gained through living with health and illnesses, and receiving healthcare.^3^ ‘Experiential knowledge arises when these experiences are converted, consciously or unconsciously, into a personal insight’.^4^ Personal experiences in the form of narratives are commonplace in everyday life. We tell our companions stories about ourselves and others all the time, for a myriad of purposes, including the building and sustaining of interpersonal relationships or the management of misfortune. In a research setting, a personal story may help put a healthcare intervention into the context of what it is like to be on the receiving end of care. The story is valuable in both settings, for different reasons and with different meanings.

Public collaborators are asked to contribute to work, which is carried out within the research community.^5^ In this community, knowledge is practised within a social hierarchy. As outsiders, public collaborators cross an epistemic boundary between everyday language and technical scientific terminology. Stories are not part of conventional scientific discourse and within it, stories and ‘anecdotes’ have less value than ‘facts’ and ‘findings’.^6^, ^7^ Public collaborators also cross a *physical* boundary, when research meetings are held at a university.

Experiential knowledge in research might therefore be considered a ‘boundary object’. This is ‘an analytic concept of those scientific objects which both inhabit several intersecting social worlds … *and* satisfy the informational requirements of each of them’ (p. 393).^8^ While ‘experiential knowledge’ is not a material object, the forms it takes in research settings are similar or identical to the forms it takes in people's personal lives. However, the social meanings of it will differ across the boundary.

Carlile^9^ found that knowledge boundaries emerge between different areas of practice^10^ because knowledge is localized, embedded and invested.^9^ Research knowledge is *localized* in relation to the problems faced by researchers in a particular study or research practice; it is *embedded in research practices* because researchers apply methods recognized as reliable within their field; it is *invested* because researchers have used time and effort gaining knowledge. Achieving impact from PPI can be a challenge since public collaborators are visitors to this study space.

We propose that Fricker's^11^ conceptualization of epistemic injustice is relevant to enabling experiential knowledge to be a successful boundary object in health research. Fricker identified two aspects of epistemic injustice. ‘Testimonial injustice’ refers to ‘a wrong done to someone specifically in their capacity as a knower’ (p. 1),^11^ for example, when public collaborator's contributions are not taken seriously because they are not practitioners or researchers. ‘Hermeneutical injustice’ is when public collaborators do not have the conceptual tools to interpret their experiences of healthcare or to contribute their experiential knowledge, for example, when professionals use research jargon.

If health research is to benefit from experiential knowledge, public collaborators need testimonial justice. What they say needs to be taken as worthy of influencing the research.^12^ Sociologists have had a longstanding interest in people's everyday ideas about health and illness.^13^, ^14^, ^15^ These ideas have been explored in the context of public health and healthcare. Recent ethnographies of PPI have led to a deeper understanding of the processes involved.^16^, ^17^ In this study, we investigate the contribution of experiential knowledge to research, applying the theory of epistemic injustice.

## 2 METHODS

This study's aim was to increase understanding of how experiential knowledge is used in research settings. Its objectives were to build a working definition of experiential knowledge, use this to analyse data and identify practical applications of our findings to inform PPI practice. We conducted a qualitative study of the involvement of (i) audio transcripts of meetings between researchers and public collaborators and (ii) interviews with public collaborators and researchers.

Ethical approval was granted by the University of Exeter College of Medicine and Health Research Ethics Committee (Nov16/D/100∆8).

### 2.1 Study sample and data collection

This study was based on a convenience sample of three projects (Table 1). These projects were led by researchers who are not authors of this paper. Public collaborators were paid for their time and travel and had a named administration contact for practical help.

**Table 1:** Details of the study meetings and interviews

Project	Meeting type	Meeting purpose	Setting	Meeting attendees	Public collaborators' previous involvement in research	Interviewees from the meeting
*Antenatal* Aim: To develop an intervention for pregnant women	First of a series of PPI advisory group meetings to inform the study.	To discuss findings from the study's literature review.	Community centre playroom.	Two researchers, one PPI facilitator (author of this paper), six mothers who had recently given birth, with babies and toddlers present.	All were new to patient involvement. They volunteered after researchers approached an existing toddler‐parent group.	Two public collaborators and two researchers.
*Data set* Aim: To develop a new prognostic model for risk estimation	First research project team meeting, held monthly.	To discuss and make decisions on the study protocol in response to peer review.	University meeting room, round‐table set‐up.	Eight researchers, one PPI facilitator (author of this paper), two public collaborators.	Previous involvement in research or NHS committees.	Two public collaborators and one researcher.
*Screening* Health Technology Assessment of a screening interven‐tion	First of three one‐off workshops with public collaborators. All workshops followed the same format and purpose, but with different public collaborators.	To identify relevant outcomes for the protocol and discuss the relevance of health inequalities to screening uptake.	University meeting room, tables set up for three small groups.	Two researchers from the project team, one researcher observing the meeting, one PPI facilitator, nine members of the public. Both the observing researcher and the PPI facilitator are authors of this paper.	Two were new to involvement, two had limited experience of it, and five had previous experience of being involved in research.	Two public collaborators and one researcher.

Abbreviations: NHS, National Health Service; PPI, patient and public involvement.

The public collaborators were all potential beneficiaries of the interventions being researched. For each project, the first meeting with public collaborators was selected for analysis. The meetings would have happened irrespective of people's consent to audio recording; however, all participants gave written consent.

Six public collaborators were invited and consented to participate in face‐to‐face individual interviews. We asked how they were (or not) able to contribute their own experiences, and whether they edited their experiences to fit the meeting context. Participants were offered a £10 voucher for taking part. Five researchers were invited to interviews and four consented to face‐to‐face individual interviews. We explored their perceptions of public collaborators' contributions. Interviews were conducted by authors who had not been involved in the projects.

Interviews lasted about 1 h in a place chosen by the interviewee. Interviews were transcribed using pseudonyms.

### 2.2 Data analysis

Data analysis was informed by Green and Thorogood.^18^ First, we conducted a preliminary and inductive analysis of the three meeting transcripts to develop a coding framework for experiential knowledge (Table 2). Once the framework was agreed upon, each transcript was coded in NVivo 11 by two authors. One author who had not been present at any of the meetings (N. B.) coded all three meeting transcripts.

Second, we inductively developed codes for the interview transcripts, using a similar process. All interviews were read to inform the framework. Once the coding framework was finalized, each transcript was coded independently by two authors. The coding framework was adjusted after the first round of double coding, and data were recoded with the new framework (Table 3).

In the third phase, we developed coding summaries by examining the data within each code. This allowed us to compare findings across meetings and interviews. All authors were involved in writing the summaries and selecting quotes to illustrate the findings. The quotes below were chosen due to their illustrative power, drawn from all data sources (see Box 1 for reflexivity considerations).

BOX 1. **Reflexivity**

**Table:** 

Reflexivity context: The authors include four members of the NIHR Applied Research Collaboration South West Peninsula (PenARC) PPI team and one member of the Peninsula Public Engagement Group (PenPEG). Three authors supported involvement at the meetings within the study's data set. They did not conduct interviews with participants from these meetings.
Reflexivity considerations:
The author team could be insufficiently critical of own or colleagues' practices. Public collaborators may be unwilling to criticize researchers' work.
Reflexivity activities:
At study inception we conducted a team reflexivity exercise.^19^ All authors wrote a reflective statement about their interest in the topic, how they expected to influence the study, their expectations and foreseeable obstacles. Discussion of statements did not reveal any major disagreements or conflicting aspirations, but emphasized the team's wish to improve their own practice

### 2.3 Findings

Below we elaborate on our *data‐informed* definition of experiential knowledge. We then present the three themes. *Equal treatment* ensures that public collaborators' contributions are taken seriously; *professionalism* shows the work that public collaborators do to be taken seriously; the final section illustrates the *epistemic impact* of experiential knowledge in situations of epistemic justice.

### 2.4 Experiential knowledge

Our data‐based definition states that experiential knowledge includes both own and others' lived experiences, in the forms of opinions, facts and media information. This knowledge is shared through narrative mediums: stories, seeking clarification, answering questions, commenting and references to own roles within the research space. Below, the public collaborator conveys knowledge of others' experiences through the medium of a story, while connecting this knowledge to the consideration of screening uptake, thereby making meaning of this story for the research setting.

Public collaborator: I remember a lady who used to live near me who was diagnosed, she was in her 50s, and she was diagnosed with some type of bone cancer and at the time they gave her 6 months to live. And she said this isn't going to beat me. I'm not having this. Twelve years later she passed away …. but it would depend on the individual person. Whether they think ‘oh well I've got it ‐ I will just give up now’ or whether they are determined they are going to get through it. (Screening meeting)

Seeking clarification was another medium through which public collaborators contributed their knowledge, drawing on their own experience, lack of knowledge or referencing other people's experiences. Comments included views, statements and opinions about what was being said in the meeting. Lastly, public collaborators answered questions about their behaviour, views or experiences.

In the interviews, experiential knowledge was contrasted with technical or academic knowledge and sometimes referred to as ‘ordinary’ views, beliefs, opinions, convictions, reflections and impressions of something. Some public collaborators said they shared their experiential knowledge in a different way at research meetings than when speaking to friends or family. They might share their experience but not their emotion, or they might think carefully about which parts of their experience corresponded with what the researchers asked for, and only share this. Some public collaborators said they shared things at research meetings that they would not have shared with family and friends.

‘… it was describing our experiences mainly and then we moved to kind of maybe how we might have received that information in a better way or what might have helped us to have a better understanding of it. … the team were presenting us with material and apps at one point and we would then, you know, look at those, think about how they might fit into the picture and then give our feedback about, you know, what we thought about them, how useful they were’. (Public collaborator, Antenatal project, interview)

### 2.5 Equal but different

Guidance on PPI emphasizes the value of different kinds of knowledge.^20^, ^21^ Feeling a full and equal member of the team was important to public collaborators, although there were nuances in how public collaborators spoke about this. In the data set project public collaborators said they felt more equal to researchers than in previous studies. In the Antenatal project, they focused on the importance of discussions being relevant to their situation. Public collaborators in the Screening project emphasized the importance of feeling listened to. Equality was hard to achieve because of the lack of day‐to‐day contact between public collaborators and researchers, but premeeting briefings and conscientious chairing were ways of addressing this. Researchers and public collaborators valued involvement more when they were on an equal footing.

‘And so it was just this thing of treating you as an equal around the table. So in a sense not treating you as a special person, not treating you as a different person, so you have someone who is a specialist in statistics, you have someone who's a specialist in trials management, you have someone who's a specialist in health economics, you have someone who's a specialist in public and society, and that's all you are, you're a different specialist in the room’. (Public collaborator, Datasets project, interview)

We observed a paradox: public collaborators want to be equal but they also want a distinct treatment, such as considered facilitation, premeetings and extra information. It may be that what public collaborators really want is equal treatment in terms of how their contributions are received, in other words, testimonial justice. Aside from common politeness when welcoming any collaborator to meetings, the ‘distinct treatment’ may be seen as creating the conditions in which testimonial justice flourishes; making public collaborators feel accepted in what may be unfamiliar environments.

These enabling conditions are enhanced by meeting facilitation, which can help to manage group dynamics, encourage quiet people to speak or reduce ‘off topic’ contributions. Public collaborators said that facilitation was important to bring both sides together in a constructive way.

‘I went to one PPI session and it was, you had to find out something about somebody else, … it broke down the barriers, you know’. (Public collaborator, Screening project, interview)

Our findings suggest that accessible rooms, the layout of furniture, ground rules, reimbursement of expenses and so on, can contribute to public collaborators feeling more equal at meetings. Good facilitation helps to create a receptive environment, and signals to the public collaborators that their contributions are valued.

### 2.6 Professionalism

Literature on involvement has brought forth the ‘professionalisation paradox’; when public collaborators require training to contribute to research, but this learning compromises their ‘lay’ status.^22^ Public collaborators in our study were more concerned about ensuring that their contributions were relevant. They spoke of the need to prepare for meetings by reading paperwork in advance, and to moderate their contributions to keep them meaningful to the topic at hand. Public collaborators talked about learning to tell the relevant parts of their stories. They are filtering what they say in recognition of the fact that they are in a different environment and need to change their storytelling to fit the new environment.

‘I do appreciate as much as I might want to say what I want to say, the researcher has also got a job to do and I don't want to, I would say to just try and keep it relevant, don't go off on a track, talk about Bob's cat or whatever else down the road!’. (Public collaborator, Screening project, interview)

In relation to hermeneutical injustice, the use of jargon is a frequent obstacle to PPI. Some researchers supplied jargon‐busters and lists of acronyms, met with public collaborators in community settings and held premeetings to consider the agenda in advance. Some public collaborators also described doing research at home after the meeting to look up terms that they did not understand.

In some cases, a public collaborator's existing skills and experience from previous employment may inform their contributions. This was seen in the Datasets project meeting:

Public collaborator: How will you be 100% satisfied that the equipment used to measure [clinical measurement] is totally comparable, I don't know if these things have to be calibrated, how many manufacturers there are, etcetera. (Datasets project meeting)

In this case, the public collaborator was using skills and experience developed in his working life to inform his contribution to health research, despite being unfamiliar with health services research methods.

Public collaborators felt that being professional did not require their story to be abandoned:

‘I know I've thought about this quite a lot about that by being professional it's about being able to weave your story throughout what you're doing so that it's relevant and everything sort of relates back to it. I don't forget my story and my experience I don't think by being professional, I just think that I'm able to get that across and relate it to whatever I need to relate it to’. (Public collaborator, Screening project, interview)

### 2.7 Epistemic impact

Researchers valued being able to test their plans or ideas by asking how these fitted with public collaborators' own experiences of services. Some researchers said that the breadth of opinions and understandings of public collaborators provides different ways of looking at familiar topics. Researchers said they needed to be open to hearing views and perspectives that differed from their own, as this can refresh their own thinking.

‘I thought their comments were particularly relevant, like things that even as a researcher we sort of thought, oh that's actually really interesting, we should've thought of that’. (Researcher, Antenatal project, interview)

Some researchers talked about having their eyes opened. This helped redress researchers' constrained mentality: when they had been working on something for so long they could not imagine how someone else would see it differently:

‘So it's that separate insight, that ability to look in and see the things we can't see ourselves because we've got our blinkers on’. (Researcher, Datasets project, interview)

Public collaborators can go further than providing fresh perspectives. PPI can challenge the ways in which researchers talk and think, and that is an important purpose.^23^, ^24^

‘So there are interesting examples of them challenging things our statistician might say and quite interesting interactions in getting [them] to come down to earth slightly and talk in a language that we can all understand. So bringing that equalisation together was quite interesting. Yes, and there's quite good interaction in the sense that, I certainly don't always understand what our statistician is saying and we sort of empowered each other to bring out into plain language that we could both understand’. (Researcher, Datasets project, interview)

The claim made by this researcher, that public collaborators were challenging what the statistician was saying, is corroborated in the meeting transcript:

Public collaborator 3: The biggest problem I had was with the data section and the, er, the comment ‘a [statistical technique] will be performed by dichotomising [the data]’ and so on. When you read it as a lay person, we struggle, it doesn't really mean a lot. And so the conversation we've just been having, whilst you said ‘is that understood’, I think I'm with you now, [statistical technique]. It's hard to follow when you're not a statistician and make any sense out of it.

Researcher 4: Right.

Collaborator 3: That's not a criticism….

…

Researcher: this was largely derived from pasting elements of the bid into a paper format … and it's absolutely up there for editing and I think plain English is an important part of that even in the scientific sections. So [Researcher 2] I think it's probably incumbent on us to just make that a bit clearer….

(Datasets project meeting)

Public collaborators may also tell researchers what to do, although they may need to do this several times before being heard. A different form of challenge is when public collaborators reveal uncertainties in clinical practice, stimulating researchers to generate evidence, which addresses these uncertainties and feedback into clinical practice. Academics acknowledged that feedback from public collaborators changed the way they thought about their work, for example by identifying unanticipated outcomes or uptake of the intervention.

Researcher: The issues that we talked about in that other sections report were very much around the participation aspect, so that we would not normally have talked about, and there's no, if you think about a trial, the sorts of research that you would do, you don't get very much information about participation rates.

Interviewer: You mean why people accept or don't accept?

Researcher: Exactly. And this allowed us, the PPI allowed us to talk about that, and say, ‘We did this exercise and this gave us information about participation and nonparticipation’.

Interviewer: A hugely important issue for a screening programme.

Researcher: Absolutely, absolutely, and, of course, in many cases you don't find about that until you've introduced the screening programme.

(Researcher, Screening project, interview)

At a general level, PPI could broaden the conversation about a whole programme of work going beyond the particular project being discussed. Involvement could change the emphasis of a project by shifting the focus from influencing professional behaviour to the consequent benefit for patients. Some of the challenges voiced by public collaborators concerned the process of involvement itself.

Public collaborator: Not wanting to be precious but that is absolutely no mention in that list of anything. PPI representatives.

(Datasets meeting)

There were differences in how public collaborators spoke about their ability to challenge at the meetings. Public collaborators in the Antenatal project felt able to speak freely, while those in the Datasets project emphasized technical jargon as a barrier. In the Screening project public collaborators had noticed that certain contributions were rejected:

‘the lead researcher, not [name], but the lead researcher didn't seem very receptive, because some of the, what was being said, and I can't remember it verbatim, it didn't seem to sit with how she felt it would go’.

(Public collaborator, Screening project, interview)

Researchers and public collaborators may have ambivalent views about the nature of the challenge, and not all of them used this language. Although some researchers claimed to have been challenged in meetings, and some public collaborators claimed to have challenged researchers, this was not always evidenced in the relevant meeting transcripts. Overall, we did not identify examples of strong challenges, although we did find examples where public collaborators asked questions. It may be that any query or alternative perspective feels challenging to some researchers. Public collaborators are aware that challenges need to be made carefully, and in the interviews, they may have claimed to have been more challenging than they were in practice.

## 3 DISCUSSION

The lower value attached to experiential knowledge and personal stories in scientific settings, because they have crossed the boundary from everyday life, creates the potential for epistemic injustice. This study has shown what experiential knowledge contains in terms of personal knowledge of own and others' experiences. We have also shown the mediums that this knowledge is shared through. Our study suggests PPI impact relies on meetings that provide a context of epistemic justice, in which public collaborators' experiential knowledge is valued.

Experiential knowledge has been defined as ‘pragmatic rather than theoretical or scientific, … oriented to the here‐and‐now‐action … and holistic’ (p. 449).^3^ Experiential knowledge spans embodied knowledge gained through personal lived experiences, and empathetic knowledge gained through long and intimate associations with people cared for.^25^ A related term is ‘lay knowledge’, defined as the articulation of meanings people ascribe to health, illness, disability and risk.^13^, ^14^ Our study adds to these works by emphasizing what it contains and how it is shared in the context of health research.

Popay et al.^13^ investigated how lay knowledge is formed, and what this can do for research knowledge generation. They connect people's active use of narrative in meaning‐making with theoretical works by Somers^26^ and Finch^27^ on space. Norms and obligations are shaped through narratives of explanation, and these narratives become part of the action itself.^13^, ^27^ Somers' concept of ‘relational settings’ (relations between people, narratives and institutions),^26^ illustrates how narrative constructions are situated within time and place.

As carriers of social practices through time^28^ public collaborators' contributions reflect what they have experienced in terms of using their physical body (bodily activities), objects they have interacted with (e.g., medicines, food, transport), language used and thinking (routinized ways of understanding the world). Knowledge here includes knowing how, ways of wanting and ways of feeling. Knowledge is informed by the social practices we engage in as humans, embedded in complex social and physical structures. Epistemic justice relates to our capacity as a giver of this knowledge, a capacity which is intrinsic to human value.^11^, ^29^

‘Experiential knowledge’, as observed and described in three research meetings, speaks to this intersection of forms and sources of personal experience that together become knowledge. ‘Experiential knowledge’ brings to research public collaborators' lived, felt and embodied knowledge as practice.

In this study, public collaborators were conscious that in researcher‐led meetings they had crossed a boundary, and some adapted their contributions accordingly. The mediums that facilitated boundary‐crossing were stories, comments and questions. For example, we observed in our meeting data and researchers told us in interviews, that naïve questions can trigger a deeper understanding by researchers of the work they do. These questions can be asked on either side of the scientific boundary, but their function in the research meeting is different than in personal or social situations.

As highlighted by Fox,^30^ boundary objects may have positive or negative social meanings and clearly, patients' stories have the potential to be seen as disruptive of scientific discourse. A successful boundary object needs to have a shared syntax to represent knowledge and be a means for individuals to specify and learn about differences and dependencies across a given boundary.^9^ We propose that in PPI, experiential knowledge can be a successful boundary object that enhances researchers' understanding of their field and how to research it. Our data also suggest that the extent to which experiential knowledge is a successful boundary object depends on whether public collaborators experience epistemic justice.

In research settings, the personal experiences of public collaborators may challenge the normal requirements for objectivity, brevity and representativeness. Thus, there are challenges in using experiential knowledge as a boundary object in scientific settings; individual public collaborators turn their narratives into ‘useful’ contributions to a scientific discussion. How they do this depends on how researchers facilitate or allow for public collaborators' contributions. Hutchison et al.^31^ propose that public collaborators often experience testimonial injustice in the health research context because all parties fail to recognize what they have to offer.

Newbigging and Ridley^29^ identify two main approaches for addressing epistemic injustice. The first is to increase the epistemic humility of the listener, that is their capacity to be virtuous listeners to create awareness of injustices that are experienced. The second includes efforts to strengthen the voice of people who are experiencing injustice, including their agency to change understandings of illness and distress. In its ideal form, PPI in research would encapsulate both these approaches.

Contextual factors, such as community settings and pre‐meetings, can create a space that enables public collaborators to settle into their role and feel reassured that their experiential accounts are welcomed. Renedo and Marston^16^ drawing on Massey^32^ write about the material dimensions of PPI: ‘The spatial is simultaneously a sphere of constraints and opportunities where “there are always connections yet to be made” and interconnections that “may or may not be accomplished”…’ (p. 490).^16^ Physical spaces and meeting structures can allow for (or prevent) emotion and personal experiences to be shared, heard and influence decisions in research. A stronger attention to the meeting space can help public collaborators to share more fully their perspectives, drawing on their whole experience rather than individual parts.

The researchers we interviewed appreciated the value of experiential knowledge in broadening their, sometimes, constrained perspectives, bringing insights that would otherwise have been overlooked. Similarly, Renedo et al.^33^ found that in spite of hierarchical structures, collaboration can be facilitated by an emphasis on nonhierarchical relationships where patients have roles of equal importance to others on the study team. For the public collaborators, hermeneutical injustice due to jargon seemed to be a persistent problem, sometimes attended to by researchers, and sometimes not, leaving public collaborators to do extra work. Our study suggests that considerable efforts are needed to address the hierarchy, especially by researchers. It is helpful but not sufficient to focus on good facilitation, chairing and induction of public collaborators into the space. Active listening skills are also needed by researchers.

There was little evidence that researchers found the contributions of public collaborators to be disruptive or inappropriate. Possibly this was because the public collaborators adapted to the unfamiliar environments by choosing their words carefully. Both researchers and public collaborators said that PPI challenged researchers. Our data suggest that such challenges might be experienced as strong even when they are raised in a careful and diplomatic manner. All these meetings were led by researchers, so we do not know if experiential knowledge is received differently in meetings led by public collaborators, or with different aims.

Our findings suggest that there is a need for the considered treatment of public collaborators when meetings are held in the research space. This treatment is there to create the conditions for epistemic justice. In considering the virtue of hermeneutical justice, Fricker^11^ discusses ways in which this may be fostered. She invokes the idea of a virtuous hearer who develops a reflexive sensitivity to avoid making prejudicial judgements about what others are saying. This may involve an awareness of the different social identities of the hearer and speaker. In the context of research, the different social identities of public collaborators and academic researchers can create credibility gaps, which can be bridged by facilitating an inclusive climate. This expands our understanding of epistemic justice in institutional settings, and the work required to achieve a collective virtuous hearing.

## 4 STRENGTHS AND LIMITATIONS

The strengths of this study lie in the complementary sources of data from three contrasting projects and the active involvement of public collaborators all the way through from the original proposal to writing this paper. The limitations are that the team researched its own practices, and the study was based within a single institutional context. The involvement support was similar across the projects but the projects themselves were very different and led by different researchers. A similar study conducted elsewhere might have surfaced different concerns, for example, if the public collaborators did not have one named contact for practical queries, or if the researchers at the meetings were less committed to involvement.

The specific institutional context is also a strength. It shows that in spite of considerable efforts to support public contributors to attend and participate in meetings there were still barriers to experiential knowledge being shared and taken seriously.

## 5 CONCLUSION

This study aimed to increase understanding of how experiential knowledge is used in research settings. It found that public collaborators brought experiential knowledge in the form of their own personal experiences, experiences of others and knowledge gained through occupation or media. Public collaborators were mindful of their lack of research knowledge, and this awareness was brought to the meetings, as well as their knowledge that a patient's expertise is usually granted lower status than a professor's. Public collaborators' knowledge of their own personal lives and how this differs from research knowledge, influenced how they negotiated epistemic justice for their narratives as they crossed the boundary into research. While researchers generally were open to PPI and appreciated contributions by public collaborators, there were barriers to experiential knowledge being taken seriously. This study highlights that avenues for addressing these include special arrangements to help public collaborators contribute, as well as equal reception of their contributions within the research space.

**Table 2:** Experiential knowledge: Coding framework from the meeting transcripts

Category	Code	Definition
1. Roles	1.1 Reference to public collaborators' own role	A statement, story, comment or other utterance *comes from a public collaborator* and includes a reference or alluding to the role that the person has as a public/patient/carer collaborator. For example, by saying ‘I don't want to be difficult’, ‘this isn't a criticism’, ‘can I just ask’, and so forth.
2. Clarification—Public collaborator asks *clarifying* questions which are:	2.1 Clarification supported by own experience	A public collaborator asks a question that refers to, or is clearly anchored in, their own experience. It needs to be explicit that the experience referred to is their own. For example, ‘Would that work? Because when I tried it didn't really help me’.
	2.2 Explicit reference to own lack of knowledge	Absence of knowledge—could be technical or experiential—the public collaborator explicitly refers to own lack of knowledge, for example, I don't know, I'm not sure and so forth.
	2.3 Clarification supported by one's own understanding of the topic	A public collaborator asks a question that is anchored in their knowledge about the topic. Their topic knowledge must be clearly referred to, for example, ‘So if I understand you correctly, clinical effectiveness is found when you compare this new treatment to what's currently delivered by GPs?’.
	2.4 Clarification with reference to other people's perspectives/lives including popular culture	A public collaborator asks a question on behalf of others, either because they are prompted or because of own initiative. For example, ‘we don't seem to have a problem with X‐raying vast amounts of people umm exposing them to radiation for breast cancer screening just wondering why we would consider it for this?’.
	2.5 Clarification, unsupported	A public collaborator asks a question but it is not clear what the question is arising from, beyond it being asked to clarify something. This could be naïve questions, for example, ‘What is clinical effectiveness?’ and this could be asked more elaborately, for example, ‘When you told us about the study and the treatment, you mentioned clinical effectiveness and I don't understand this word, what is clinical effectiveness? Has it got anything to do with clinical practice?’.
3 Stories—Public collaborator tells stories	3.1 Personal story	Stories related to themselves.
	3.2 Distancing story	Shares a story but they are using nouns that generalize the story, for example, words like ‘one’ instead of ‘I’, or ‘people’ instead of ‘we’.
	3.3 Others' story	Shares a story about a friend, family member, or something they witnessed or were told. This can include observed stories about something a public collaborator has seen, for example, someone's treatment by a nurse, and representative stories where the public collaborator speaks on behalf of others and may be relating stories from, for example, a patient group they belong to.
	3.4 Concern	Shares a concern or raises a question that relates to their own experiences, for example, but not limited to asking for advice or bringing up a problem with an existing service.
4. Answering a question	4.1 Answering questions	A public collaborator answers question about his/her own behaviour, views or experience directly. This is brief, can include details of experience but is shorter than a story.
5. Commenting: *Comments* are not stories, but views/statements/opinions on what's being said in the meeting. The subcodes in this category mirror the subcodes in category 2 (clarification), see this set for full definitions.	5.1 Comment supported by own experience	Connecting a personal experience with the project. It needs to be explicit that the experience referred to is their own.
	5.2 Comment with explicit reference to own lack of knowledge	Absence of knowledge—could be technical or experiential—the public collaborator makes a comment and explicitly refers to his/her own lack of knowledge.
	5.3 Comment with reference to the understanding of the topic	A public collaborator makes a comment anchored on their knowledge about the topic. Their topic knowledge must be clearly referred to.
	5.4 Comment with reference to other people's perspectives/lives including popular culture	A public collaborator makes a comment on behalf of others.
	5.5 Unsupported comment, which could include an opinion	
6. Researcher's input	6.1 Researcher experiential knowledge	Researcher shares personal experience.

**Table 3:** Coding framework from the interview transcripts

Code	Definition
7. Experiential knowledge	A public collaborator refers to what they bring to the research meetings. This is a text that in some way corresponds to our definition of experiential knowledge (stories, comments, clarifications), including reference to lack of knowledge. This ‘experiential knowledge’ code relates to people's knowledge of the research topic (not knowledge of involvement or research).
8. How do patient and public representatives address the obstacles they encounter?	How public collaborators address obstacles, includes obstacles and descriptions of how to overcome obstacles.
9. How do researchers address obstacles they experience when involving patients and members of the public in research?	How researchers address obstacles, includes obstacles and descriptions of how to overcome obstacles.
10. How do researchers perceive the value of experiential knowledge?	Text contains a value judgement, for example, ‘good’, or ‘perhaps it was a bit narrow’. Use this code also when the researchers talk about no value or lower value of involvement.
11. How do researchers describe public collaborators' role?	Text from researchers that clearly relate to how they see the role of public collaborators in research.
12. What does involvement ‘good practice’ look like, from the perspective of involved patients/public members and researchers?	Text contains a value judgement, words that evaluate the described practice, for example ‘I really liked that’, or ‘in this way they clarified to me’.
13. What does involvement ‘poor practice’ look like, from the perspective of involved patients/public members and researchers?	Text contains a value judgement, words that evaluate the described practice, for example, ‘this was quite difficult because they didn't explain’, or ‘I wasn't sure what they meant’.
14. (How) does the contribution of experiential knowledge affect the design or conduct of research studies?	Text that speaks about changes in the research made as a result of the patient and public involvement.

This study shows that considering experiential knowledge as a boundary object helps to clarify what public collaborators bring to research settings. It also shows that efforts to create epistemic justice can enable the impact of PPI in research. Creating conditions for active participation remains a challenge despite an emphasis on this by research funders. Future studies are needed to test specific activities or actions that create epistemic justice at meetings where experiential knowledge is invited.

## AUTHOR CONTRIBUTIONS

**Kristin Liabo**: Conceptualization; data curation (lead); formal analysis; investigation (lead); methodology; project administration (lead); resources (lead); supervision; visualization; writing – original draft; writing – review and editing; supervision. **Emma Cockcroft**: Conceptualization; data curation; formal analysis; investigation; methodology; project administration; software (lead); visualization; writing – review and editing. **Kate Boddy**: Conceptualization; formal analysis; investigation; methodology; visualization; writing – review and editing. **Silvia Bortoli**: Conceptualization; formal analysis; investigation; writing – review and editing. **Leon Farmer**: Conceptualization; formal analysis; visualization; writing – review and editing. **Nicky Britten**: Conceptualization (lead); formal analysis (lead); investigation; methodology; project administration; visualization; writing – original draft (lead); supervision (lead).

## CONFLICTS OF INTEREST

All authors of this paper have initiated and facilitated public involvement in research over a period of many years, no other conflicts of interest declared.

## Data Availability

The original data are not shareable due to the remit of ethical approval granted.

## References

[1] Oliver M . Changing the social relations of research production? Disabil Handicap Soc. 1992;7(2):101‐114.

[2] Chalmers I , Bracken MB , Djulbegovic B , et al. How to increase value and reduce waste when research priorities are set? Lancet. 2014;383(9912):156‐165. 24411644 10.1016/S0140-6736(13)62229-1

[3] Borkman T . Experiential knowledge: a new concept for the analysis of self‐help groups. Soc Serv Rev. 1976;50(3):445‐456.

[4] Caron‐Flinterman FJ , Broerse JEW , Bunders JFG . The experiential knowledge of patients: a new resource for biomedical research? Soc Sci Med. 2005;2005(60):2575‐2584. 10.1016/j.socscimed.2004.11.023 15814182

[5] Pyrko I , Dörfler V , Eden C . Thinking together: what makes communities of practice work? Hum Relat. 2017;70(4):389‐409. 10.1177/0018726716661040 28232754 PMC5305036

[6] Popper K . Conjectures and Refutations: the Growth of Scientific Knowledge. Routledge and Kegan Paul; 1963.

[7] Murad MH , Asi N , Alsawas M , Alahdab F . New evidence pyramid. BMJ Evid Based Med. 2016;21:125‐127. 10.1136/ebmed-2016-110401 PMC4975798 27339128

[8] Star SL , Griesemer JR . Institutional ecology, ‘translations’ and boundary objects: amateurs and professionals in Berkley's museum of vertebrate zoology, 1907‐39. Soc Stud Sci. 1989;19:387‐420. 10.1177/2F030631289019003001

[9] Carlile P . A pragmatic view of knowledge and boundaries: boundary objects in new product development. Organ Sci. 2002;13(4):442‐455. http://www.jstor.org/stable/3085976

[10] Wenger E . Communities of Practice: Learning, Meaning, and Identity. Cambridge University Press; 1998.

[11] Fricker M . Epistemic Injustice: Power and the Ethics of Knowing. Oxford University Press; 2007.

[12] Knowles SE , Allen D , Donnelly A , et al. Participatory codesign of patient involvement in a learning health system: how can data‐driven care be patient‐driven care? Health Expect. 2021;25:103‐115. 10.1111/hex.13345 34668634 PMC8849220

[13] Popay J , Williams G , Thomas C , Gatrell A . Theorising inequalities in health: the place of lay knowledge. Sociol Health Illn. 1998;20(5):619‐644.

[14] Popay J , Williams G . Public health research and lay knowledge. Soc Sci Med. 1996;42(5):759‐768. 8685744 10.1016/0277-9536(95)00341-x

[15] Blaxter M . The causes of disease: women talking. Soc Sci Med. 1983;17(2):59‐69. 6836342 10.1016/0277-9536(83)90356-8

[16] Renedo A , Marston C . Spaces for citizen involvement in healthcare: an ethnographic study. Sociology. 2015;49(3):488‐504. 10.1177/0038038514544208 26038612 PMC4436278

[17] Komporozos‐Athanasiou A , Fudge N , Adams M , McKevitt C . Citizen participation as political ritual: towards a sociological theorizing of ‘health citizenship’. Sociology. 2016;52:744‐761. 10.1177/0038038516664683

[18] Green J , Thorogood N . Qualitative Methods in Health Research. Sage; 2004.

[19] Barry C , Britten N , Barber N , Bradley C , Stevenson F . Using reflexivity to optimize teamwork in qualitative research. Qual Health Res. 1999;9(1):26‐44. 10.1177/104973299129121677 10558357

[20] Liabo K , Boddy K , Bortoli S , et al. Public involvement in health research: what does ‘good’ look like in practice? Res Involv Engagem. 2020;6(1):11. 10.1186/s40900-020-0183-x 32266085 PMC7110674

[21] NIHR . UK standards for public involvement. Accessed June 13, 2022. https://sites.google.com/nihr.ac.uk/pi-standards/home

[22] Ives J , Damery S , Redwod S . PPI, Paradoxes and Plato: who's sailing the ship? J Med Ethics. 2013;39:181‐185. 10.1136/medethics-2012-100512 22267385

[23] Staley K . Changing what researchers “think and do”: is this how involvement impacts on research? Res All. 2017;1(1):158‐167.

[24] Liabo K . Care leavers' involvement in research: an ethnographic case study on impact. Qual Soc Work. 2016;17(1):133‐151.

[25] Abel EK , Browner CH . Selective compliance with biomedical authority and the uses of experiential knowledge. In: Lock M , Kaufert P , eds. Pragmatic Women and Body Politics. Vol 1. Cambridge University Press; 1998:310‐326.

[26] Somers MR . The narrative constitution of identity: a relational and network approach. Theory Soc. 1994;23:605‐649.

[27] Finch J . Family Obligations and Social Change. Polity Press; 1989.

[28] Reckwitz A . Toward a theory of social practices: a development in culturalist theorizing. Eur J Soc Theory. 2002;5(2):243‐263.

[29] Newbigging K , Ridley J . Epistemic struggles: the role of advocacy in promoting epistemic justice and rights in mental health. Soc Sci Med. 2018;219:36‐44. 10.1016/j.socscimed.2018.10.003 30359905

[30] Fox NJ . Boundary objects, social meanings and the success of new technologies. Sociology. 2011;45(1):70‐85. 10.1177/2F0038038510387196

[31] Hutchison K , Rogers W , Entwistle V . Addressing deficits and injustices: the potential epistemic contributions of patients to research. Health Care Anal. 2017;25:386‐403. 10.1997/s10728-016-0323-5 27277736

[32] Massey D . For Space. Sage; 2005.

[33] Renedo A , Marston C , Spyridonidis D , Barlow J . Patient and public involvement in healthcare quality improvement: how organizations can help patients and professionals to collaborate. Public Manag Rev. 2015;17(1):17‐34. 10.1080/14719037.2014.881535

